# Unstable elbow dislocations: a case report of a new surgical technique

**DOI:** 10.1051/sicotj/2016010

**Published:** 2016-04-05

**Authors:** Mark Harris, Sarah McMahon, Timothy Bishop, Jason Bernard

**Affiliations:** 1 Department of Orthopaedic Surgery, St Georges NHS Foundation Trust Blackshaw Road Tooting, London SW17 0QT UK

**Keywords:** Elbow dislocation, Surgical stabilisation

## Abstract

This case report describes our first clinical experience of a previously described new surgical technique for the treatment of unstable simple elbow dislocations. The technique utilises a central strip of the distal triceps tendon which is harvested proximally at the musculotendonous junction and left attached at its insertion on the olecranon. The strip is the passed through the olecranon fossa and attached to the coronoid process to stabilise the joint. We encountered an early postoperative complication that led to a modification of our technique and ultimately an excellent recovery of a stable pain free joint with a full range of movement.

## Introduction

The incidence of simple elbow dislocations is 5–6 per 100,000 [1]. Most simple elbow dislocations can be reduced closed with sedation and will remain reduced and stable [2]. A small proportion of simple dislocations are grossly unstable and do not remain reduced with standard non-operative treatment. We present a case report of an unstable elbow dislocation that we treated with a new surgical technique.

## Case report

**Figure 1.**
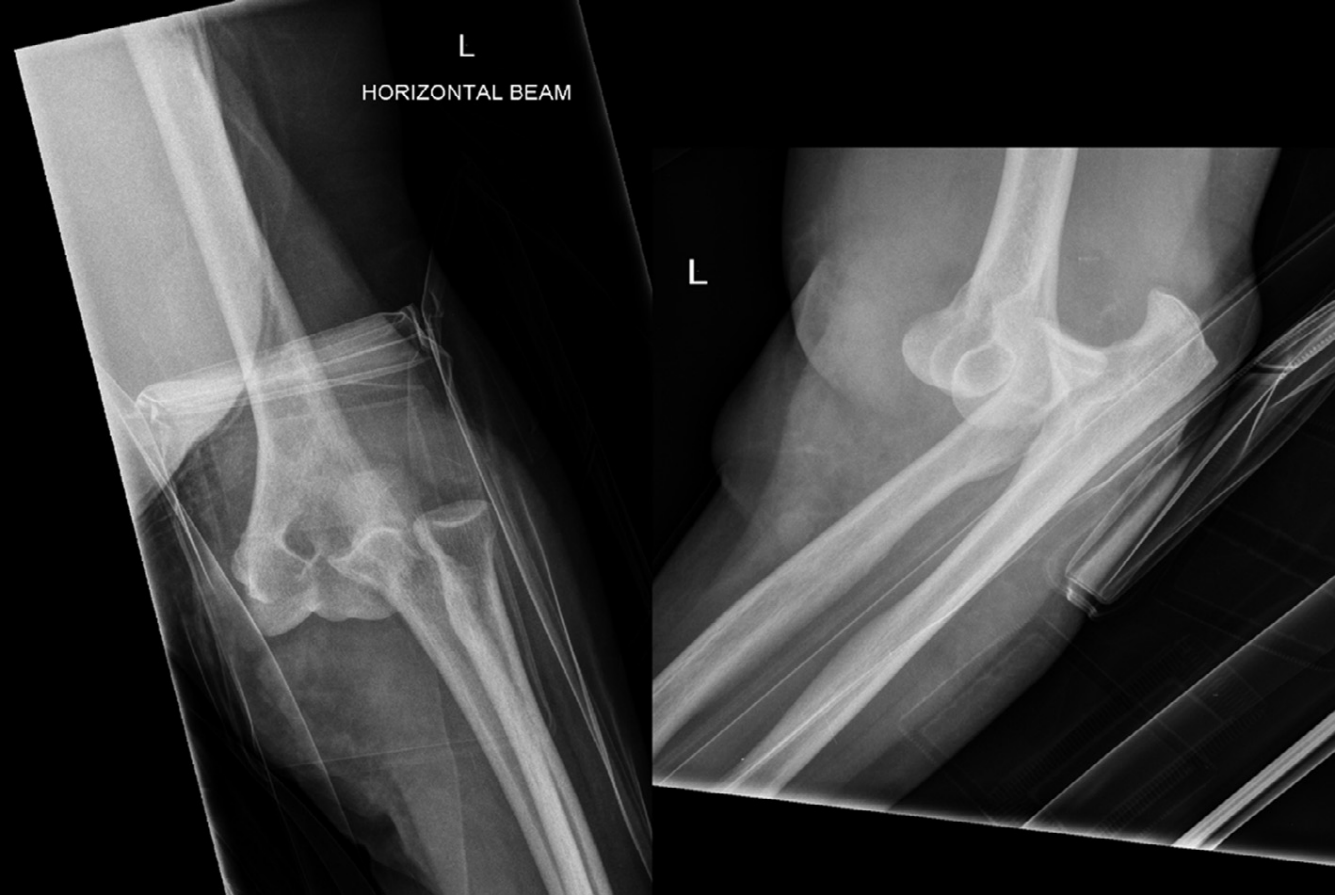
Elbow AP and lateral radiographs at presentation.

**Figure 2.**
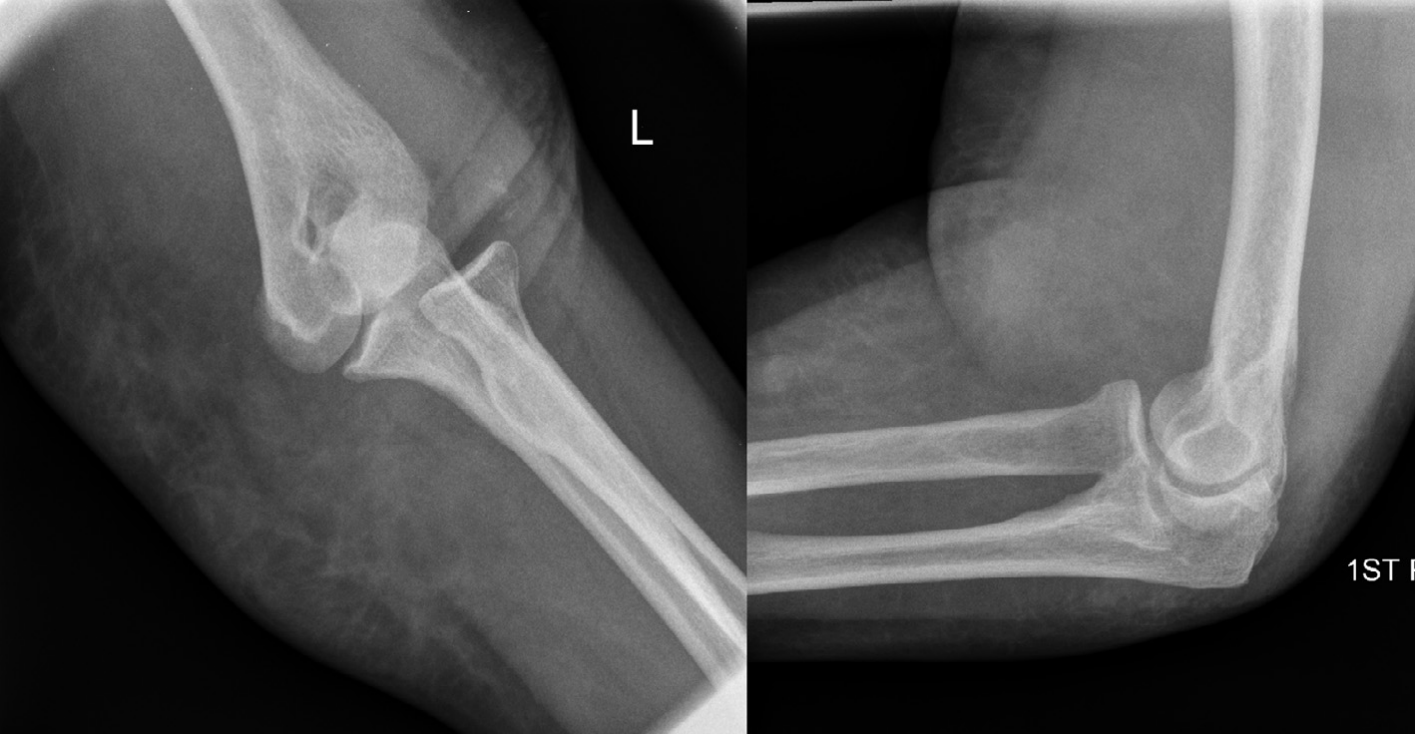
Elbow AP and lateral radiographs following closed reduction.

**Figure 3.**
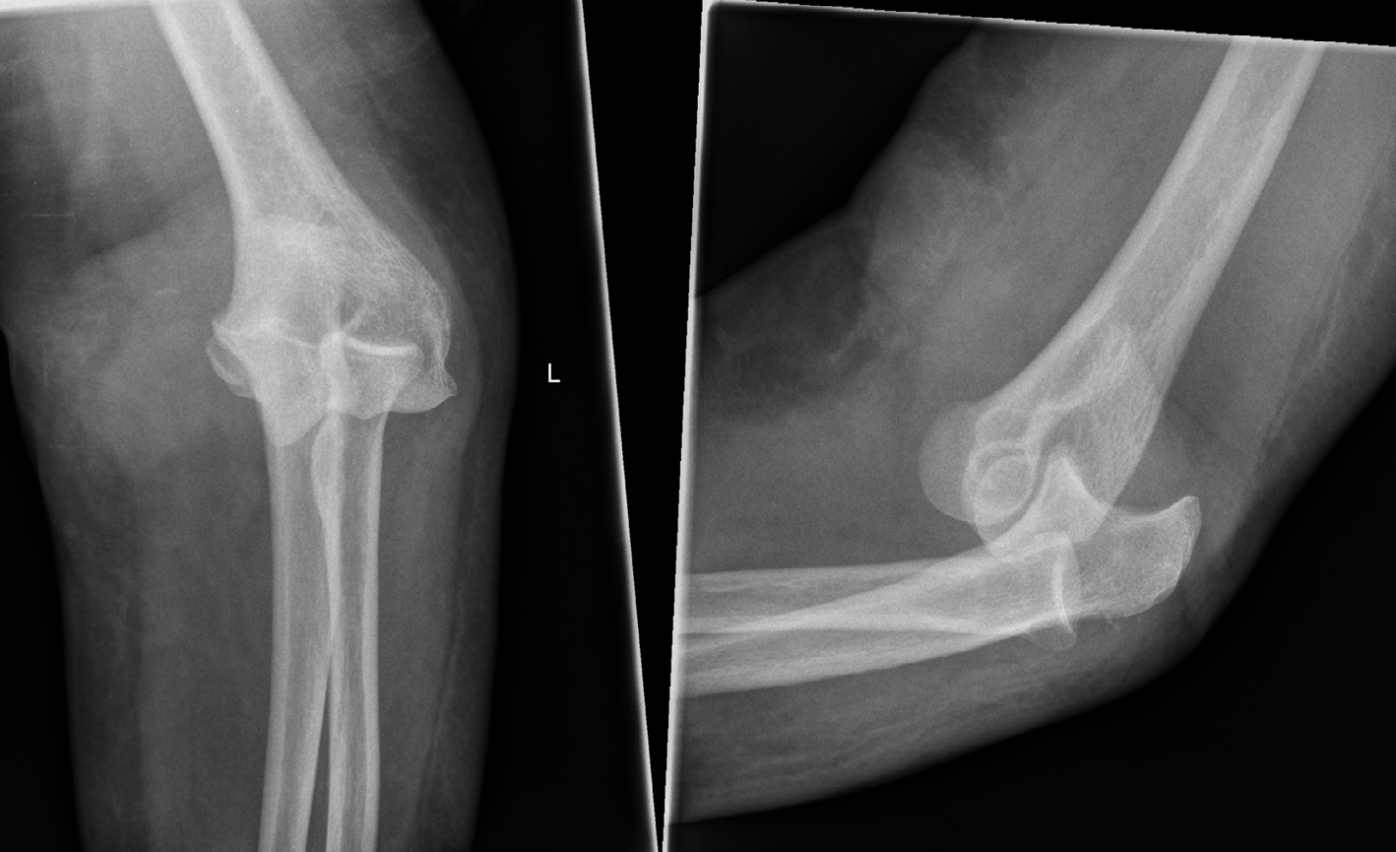
Elbow AP and lateral radiographs three weeks post injury showing dislocation.

A 73-year-old lady presented to the Accident & Emergency (A&E) department immediately after a fall from standing height with a painful deformed right elbow. Initial radiographs demonstrated a dislocation of the elbow with no associated fractures (Figure 1). The joint was manipulated by the A&E staff and the arm was placed in an above elbow plaster back slab with 90° flexion. Postmanipulation radiographs confirmed the reduction albeit with some subluxation of the radio-capitellar joint (Figure 2). The patient was discharged home. Four days later she was reviewed at the fracture clinic by a locum doctor who also did not appreciate the importance of subluxation on the post-reduction film and did not repeat the radiographs. The arm was placed in an above elbow complete cast and a review was scheduled in a further two weeks. At this review, three weeks following her injury she was in severe pain and the elbow was noted to be obviously clinically dislocated with extensive bruising and swelling. Fortunately the hand remained neurovascularly intact. Radiographs confirmed the now chronic dislocation (Figure 3). At this point the soft tissues prevented further closed reduction.

**Figure 4.**
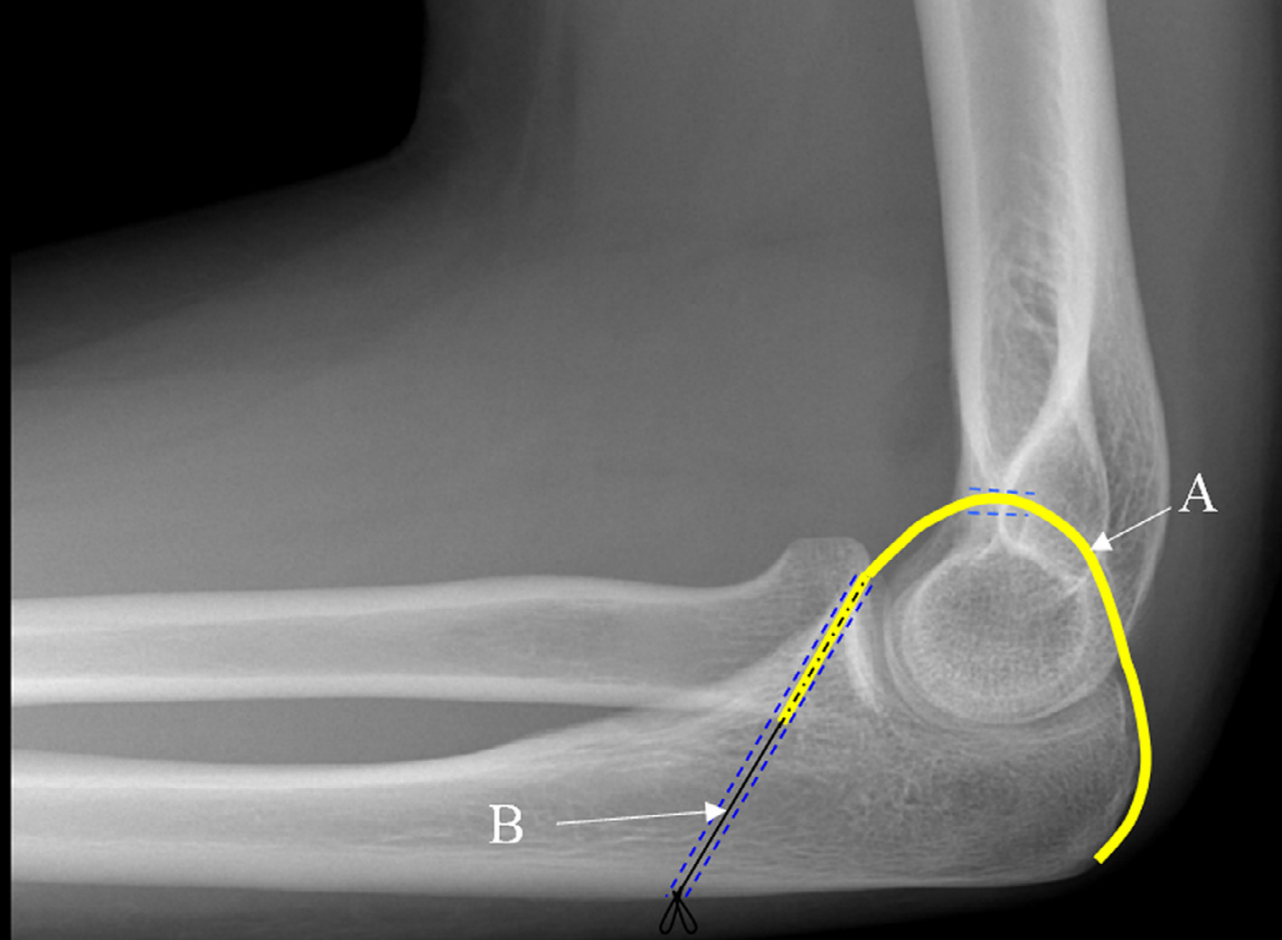
Diagram of triceps tendon (A) reconstruction and suture (B).

The patient had significant medical comorbidities: ischaemic heart disease with previous coronary stents; left ventricular failure; complete heart block with a permanent pacemaker; type 2 diabetes; a BMI > 35 and emphysema treated with long-term steroids. She was discussed in the departmental trauma meeting and due to her high anaesthetic risk, poor soft tissues and general frailty she was assessed as not suitable for our standard operative treatment with collateral ligament repair and stabilisation with a hinged external fixator. The options suggested by the consultant body at the trauma meeting were to offer either non-operative treatment (pain control medications and a brace) or percutaneous trans-articular pinning. Both these options have significant drawbacks in terms of functional outcome. We have previously described a new operative technique that we proved to be technically feasible in a cadaveric study [3]. The technique utilises the harvest of a central strip of triceps tendon which is distally based and remains attached at its insertion. The central triceps strip is passed through a fenestration made in the olecranon fossa and fixed to the coronoid process to construct a complete osseo-tendonous ring (coronoid, olecranon and triceps tendon) that holds the ulna congruent with the trochlea of the distal humerus (Figure 4). The hypothesis is that this will allow early range of motion rehabilitation and avoid the instability that would come with non-operative treatment or the stiffness associated with trans-articular pinning. After discussion of the possible benefits and risks of each option the patient elected to have the new technique.

**Figure 5.**
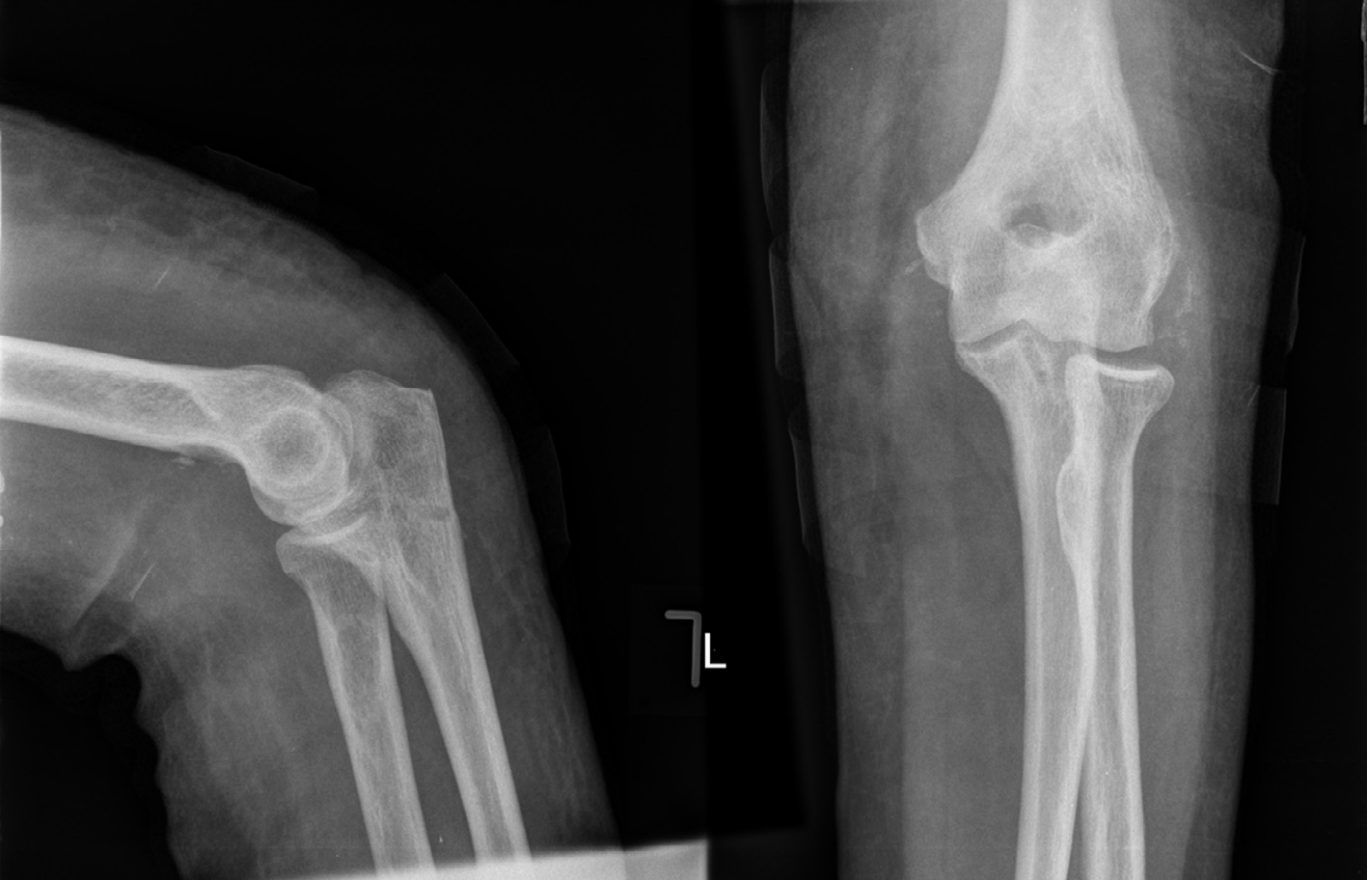
Elbow AP and lateral radiographs two weeks post initial reconstruction.

**Figure 6.**
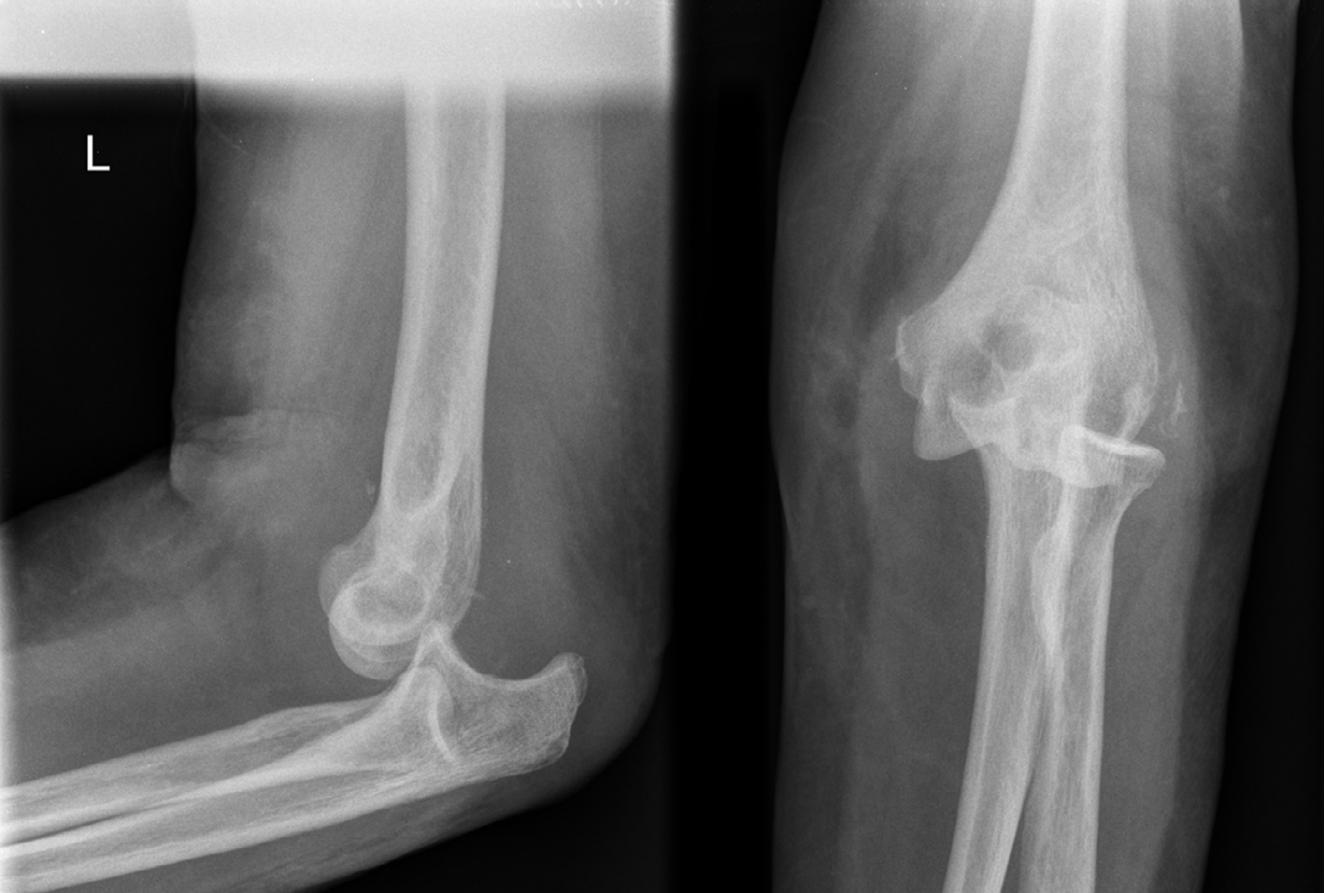
Elbow AP and lateral radiographs demonstrating failure of first reconstruction.

**Figure 7.**
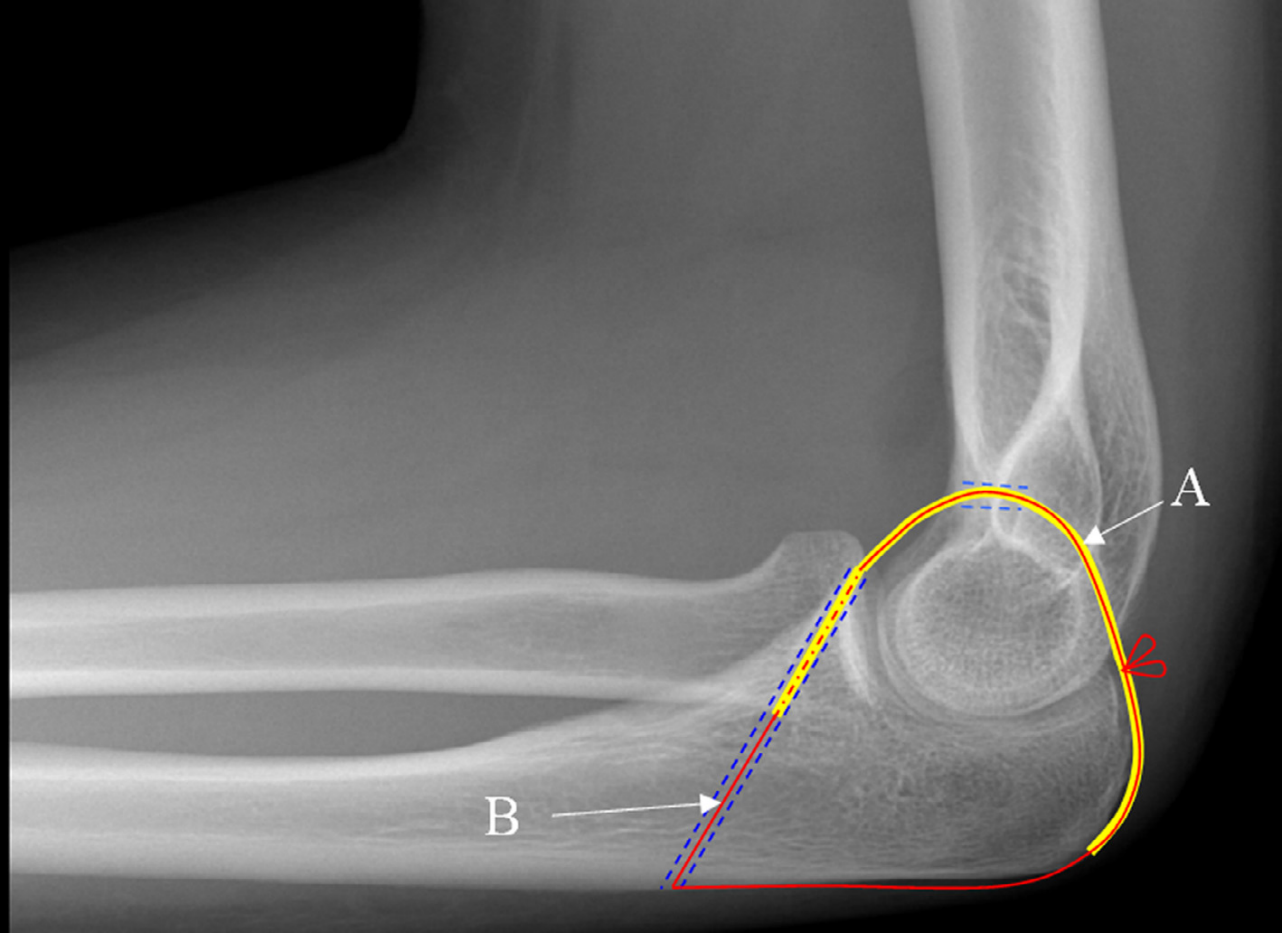
Diagram demonstrating modification of triceps tendon (A) reconstruction with extended suture (B).

**Figure 8.**
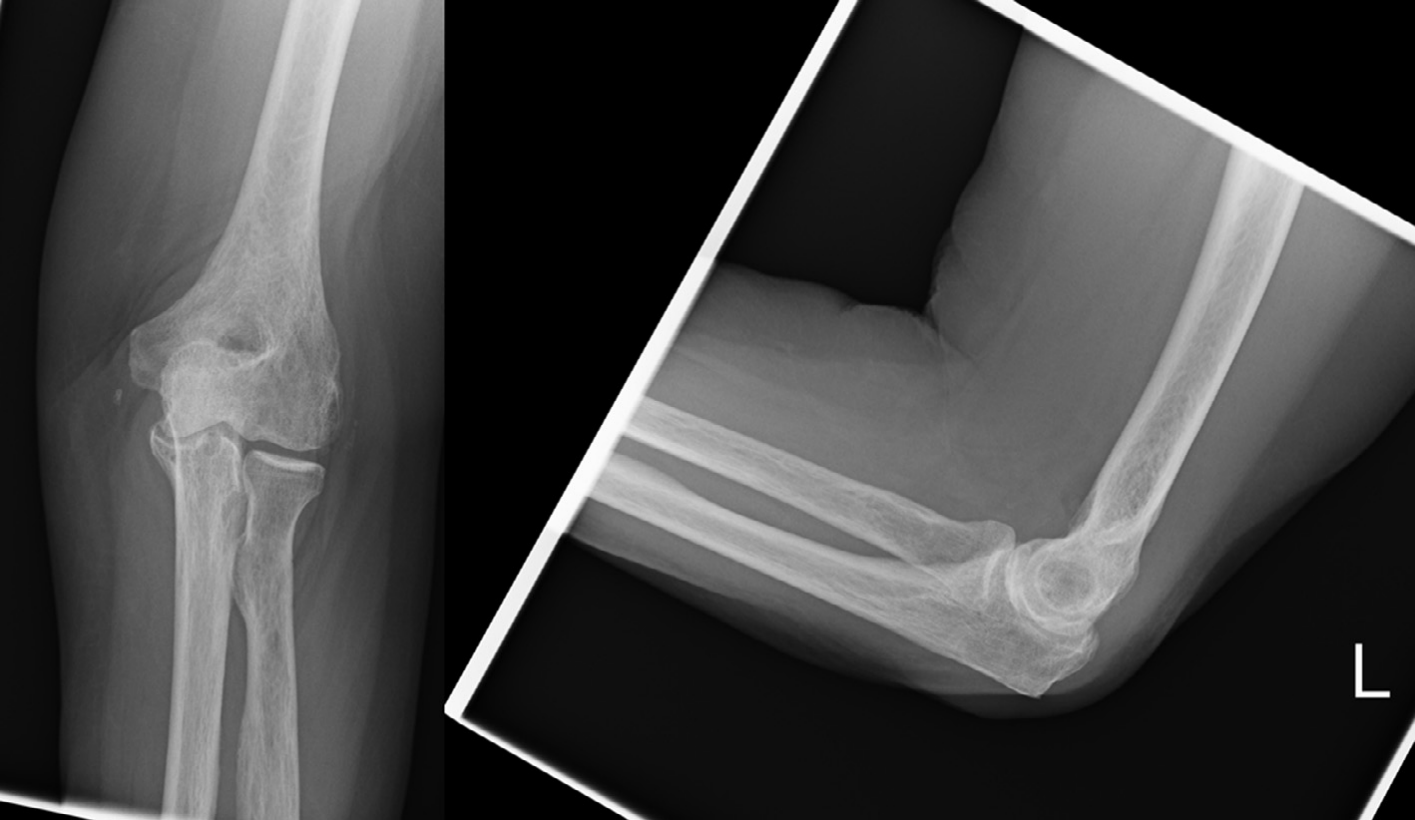
Elbow AP and lateral radiographs two years post reconstruction.

The surgery was performed under regional anaesthetic. The elbow was reduced and stressed to assess the competence of the collateral ligaments. The radial collateral ligament and both the medial and lateral ulnar collateral ligaments were completely disrupted such that the joint did not remain congruent even in flexion beyond 90°. Dislocation occurred with both external and internal rotation of the fore arm. The triceps tendon reconstruction described above was performed with an operative time of 45 min. There were no technical difficulties or complications and a congruent stable reduction was achieved (Figure 5). The patient was allowed to use the arm freely with no bracing and was discharged home on the second postoperative day. She made an excellent initial recovery. At two weeks the elbow was painfree and the wound had healed with no evidence of infection. Elbow flexion was 15–90° at two weeks and 0–110° at three weeks. Supination and pronation were normal at two weeks. She was allowed to use the arm freely including lifting and carrying as she was able. Unfortunately at four weeks following the reconstruction the patient was readmitted with a further dislocation of the elbow (Figure 6). The reconstruction was explored in the operating theatre and was found to have failed by the grasping suture in the triceps graft cutting out of the tendon. Why this occurred could not be elucidated from assessing the elbow intraoperatively. It is possible that the triceps tendon was not strong enough to resist the forces applied to the reconstruction during our initial unrestricted rehabilitation protocol. The grasping suture in the graft was revised but this time with a modification to form a complete loop from the triceps insertion through the olecranon fossa and ulna shaft back to the triceps insertion thus reenforcing the reconstruction (Figure 7). We prescribed a more conservative rehabilitation regimen following the revision. She was advised to perform active range of motion exercises with the support of a brace but no lifting or carrying of any weight. Again she made a prompt recovery. At two weeks following the revision the joint was congruent, there was no infection and elbow flexion was 10–110°. At four weeks after the revision the brace was discontinued and her range was 0–120°. Her recovery was again impressive, regaining a full painfree range of motion of elbow flexion and extension, and forearm pronation and supination. This is preserved at her most recent follow-up two years postoperatively and her radiographs confirm ongoing joint congruence (Figure 8).

## Discussion

This case report documents our experience and the outcome of the first clinical use of our novel technique for the stabilisation of a simple elbow dislocation. According to O’Driscoll et al. [4] this was a 3C injury, in that the ligamentous disruption was severe enough that the joint congruence could not be maintained with simple closed reduction and elbow flexion beyond 90°. In this scenario surgical stabilisation is indicated. Typically this involves repair or reconstruction of the ruptured collateral ligaments and joint capsule. The repair is usually protected with a brace or an external fixator. In the case we have described, we assessed her injured soft tissues as being unsuitable (due to her elevated BMI and long-term steroid treatment) for repair. We also discussed an external fixator with the patient and she was concerned she would not tolerate this. The new technique used in this case has a number of potential advantages. The triceps tendon is rarely injured in simple elbow dislocations meaning the graft quality should be good. The reconstruction is technically simple with minimal soft tissue dissection and a short operative time. There is no implanted metalwork. The recovery in the first two weeks was excellent and led us to progress to unrestricted use of the arm. This proved to be sooner than was ideal and we suspect this to be the reason for the failure of the primary reconstruction at the tendon-suture interface. The revision reconstruction used a modification to the original technique to guard against this potential weakness. As described in the case history the tendon suture was redone and extended to complete a loop around the distal humerus ensuring that the tendon-suture interface was offloaded. The subsequent recovery of elbow function compares favourably with recently published outcomes of operative treatment of unstable elbow dislocations [5].

## Conclusion

The treatment of grossly unstable elbow dislocations in elderly patients with significant comorbidities is a difficult surgical challenge. In spite of the initial failure of our new surgical reconstruction, the subsequent modified technique achieved an excellent result. This first case report establishes the grounds for further research to investigate the potential role our new technique in comparison to more traditional stabilisation techniques for the treatment of unstable elbow dislocations.

## Conflict of interest

The authors declare no conflict of interest in relation with this paper.
